# Echocardiography in duchenne muscular dystrophy: a call for consistency and standardisation of reporting

**DOI:** 10.1186/s44156-026-00130-6

**Published:** 2026-08-03

**Authors:** Lynne Williams, Sadie Bennett, Charlotte Atkinson, Daniel X. Augustine, Maria Bland, Hatty Grant, Jade Hobday, Anna Johnson, Kadhim Kadhim, Lisa Kuhwald, Chiara Marini Bettolo, Adam Kerr, David Oxborough, Liam Ring, Shaun Robinson, Jo Sopala, Cathy Turner, Chet Villa, Michela Guglieri, John Bourke, Caroline Coats

**Affiliations:** 1 Department of Cardiology, Royal Papworth Hospital, Cambridge, UK; 2 Heart and Lung Clinic, University Hospitals of North Midlands, Stoke-on-Trent, UK; 3 Cardiovascular Clinical Research Facility, University of Oxford, Oxford, UK; 4 University Hospital Southampton, Southampton, UK; 5 Department of Cardiology, Royal United Hospitals, Bath, UK; 6 University of Bath, Bath, UK; 7 Department of Cardiology Freeman Hospital, NUTH Hospitals NHS Foundation Trust, Newcastle Upon Tyne, UK; 8 British Society of Echocardiography, London, UK; 9 Department of Cardiology, Birmingham Children’s Hospital, Birmingham, UK; 10 Department of Paediatric Cardiology, Freeman Hospital, NUTH Hospitals NHS Foundation Trust, Newcastle Upon Tyne, UK; 11 Duchenne UK, London, UK; 12 John Walton Muscular Dystrophy Research Centre, Newcastle University, Newcastle Upon Tyne, UK; 13 Research Institute for Sports and Exercise Science, Liverpool John Moores University, Liverpool, UK; 14 Department of Cardiology, West Suffolk Hospital NHS Trust, Bury St Edmonds, UK; 15 Department of Cardiology, Imperial Healthcare NHS Trust, London, UK; 16 Cincinnati Children’s Hospital Medical Center, University of Cincinnati College of Medicine, Cincinnati, Ohio, USA; 17 Department of Cardiology, Queen Elizabeth University Hospital, NHS Greater Glasgow and Clyde, Glasgow, UK; 18 John Walton Muscular Dystrophy Research Centre, Newcastle University Translational and Clinical Research Institute (NUTCRI), International Centre for Life, Newcastle Upon Tyne, NE1 3BZ UK

**Keywords:** Muscular dystrophy, Cardiac dystrophinopathy, Cardiac imaging, Echocardiography

## Abstract

Duchenne muscular dystrophy (DMD) is a genetically determined, neuromuscular disorder occurring predominantly in males with a prevalence of 1:3,500 to 1:5,000 live male births. It is a life limiting condition with average life expectancy of only 28.1 years in the United Kingdom. A dilated cardiomyopathy occurs in all patients with DMD, and cardio-respiratory causes now account for about 80% of all deaths in adult patients. Transthoracic echocardiography (TTE) is a widely used non-invasive imaging modality to assess cardiac structure, chamber dimensions, and myocardial function, and so plays a central role in guiding the timely initiation and adjustment of ‘heart failure’ medications. However, obtaining high quality images in patients with DMD can be challenging, particularly after patients lose ambulation, become wheelchair reliant, and require non-invasive ventilatory support. We review the current literature and evidence pertaining to echocardiography and the assessment of cardiac function in this patient group. This article seeks to discuss the strengths and limitations of TTE in patients with DMD, recommends a standard set of the measures to be reported at all assessments, and offers consensus guidance, based on expert opinion, to allow valid comparisons of serial assessments even when performed by different TTE services.

**Supplementary Information:**

The online version contains supplementary material available at 10.1186/s44156-026-00130-6.

## Introduction

Duchenne muscular dystrophy (DMD) is a rare, X-linked, neuromuscular disorder, predominantly affecting males with a prevalence of 1:3,500 to 1:5,000 [1]. DMD is caused by deletions, duplications, or point-mutations in the dystrophin gene [2–4]. Although it can have various other multi-system effects, the predominant clinical phenotype is of a muscle-wasting disorder, characterised by progressive skeletal and cardio-respiratory muscle weakness. Although survival has improved over recent decades through the provision of multi-disciplinary care, steroid therapy, non-invasive ventilation, and the early initiation of heart failure therapy, the average life expectancy of patients with DMD in the UK is still only 28.1 years [5].

Dilated cardiomyopathy (DCM) occurs in all patients with DMD, and cardio-respiratory causes account for about 80% of all deaths in adolescents and young adults [5]. The age of first detection of left ventricular dysfunction by transthoracic echocardiography (TTE) is highly variable and dependent on the sensitivity of the measures made, but increases steadily with age, ranging from 25% in boys aged 6 years old to 59% in boys aged 10 years old [6]. Currently, there is no cure for DMD, but survival has improved progressively over recent decades due to the implementation of specialist multidisciplinary care [7, 8]. To address DMD-related DCM, the 2018 Care Considerations report [9] recommends that patients undergo regular cardiac assessment using transthoracic echocardiography ± cardiac magnetic resonance imaging (cMRI) to enable the identification of left ventricular systolic dysfunction (LVSD) in its earliest stages to guide the timely initiation of heart failure (HF) medical therapy. Regular imaging assessments are important as patients with DMD often remain asymptomatic for prolonged periods, and HF symptoms emerge at a stage at which cardiomyopathy is already advanced. Even when patients develop overt heart failure, they often present atypically and symptoms can be mistakenly attributed to intra-abdominal or respiratory causes [9].

DMD Care UK is a nationwide initiative jointly led by the John Walton Muscular Dystrophy Research Centre at Newcastle University and Duchenne UK, a leading patient charity in the United Kingdom (UK). Through specialist working groups, the project develops practical care guidelines for implementation across the UK, building consensus and securing endorsement to ensure consistency in clinical practice. In collaboration with the British Society of Echocardiography (BSE), DMD Care UK established a working group to define standards for reporting transthoracic echocardiography (TTE) in patients with DMD. In parallel, a separate expert group was tasked to develop complementary guidance for the use of cMRI, and these consensus recommendations have recently been published [10]. Together, these efforts aimed to strengthen and harmonise cardiac imaging pathways, ensuring that clinicians will have high quality imaging data on which to make clinical decisions about deploying cardiac care optimally and that patients living with DMD will receive uniform, highquality cardiac care across their lifespan.

## Why is specific echocardiography guidance needed for DMD patients?

Optimal integration of cMRI and echocardiography in the comprehensive assessment of DMD-related cardiomyopathy remains an ongoing discussion, with challenges and limitations to both imaging techniques. In the United Kingdom echocardiography remains the primary imaging modality used in routine clinical practice for these patients due to wide availability, favourable tolerability, and low cost. The BSE has previously published guidance on minimum comprehensive datasets to be obtained from TTE [11], and on imaging in suspected or known DCM [12]. However, TTE-imaging of patients with DMD presents unique challenges that require an adaptive approach. Loss of ambulation, chest wall deformities, scoliosis, respiratory dysfunction, and suboptimal positioning progressively reduce image quality [13–15], limiting the ability to obtain some of the more sensitive measures of heart function as DMD advances. What measures can still be obtained therefore are sometimes the ones least sensitive to change, and so are less reliable in guiding changes in HF therapy. In addition, variability between echocardiographers abilities to overcome these imaging difficulties can confound comparisons across serial studies [16], particularly during the transition from paediatric to adult services. Against this background, the overarching aim of these recommendations was to define a reproducible, standardised TTE dataset to be obtained at every assessment in DMD patients. By ensuring consistency in acquisition and reporting, this guidance should enable valid comparisons across serial assessments and so contribute to improved outcomes for individuals living with DMD.

## How are serial cardiac imaging results used in clinical decision making?

After a diagnosis of DMD is confirmed, the family is made aware as part of early discussions that cardiac involvement is part of the condition and not a separate diagnosis. This is as a prelude to the child undergoing a first heart assessment, ideally before the age of six years, typically comprising a standard 12-lead electrocardiogram and TTE [17]. This assessment is to confirm that the heart is structurally and functionally ‘normal’ in other respects and to provide the baseline TTE dataset on ventricular function against which subsequent studies can be compared.

**Fig. 1:**
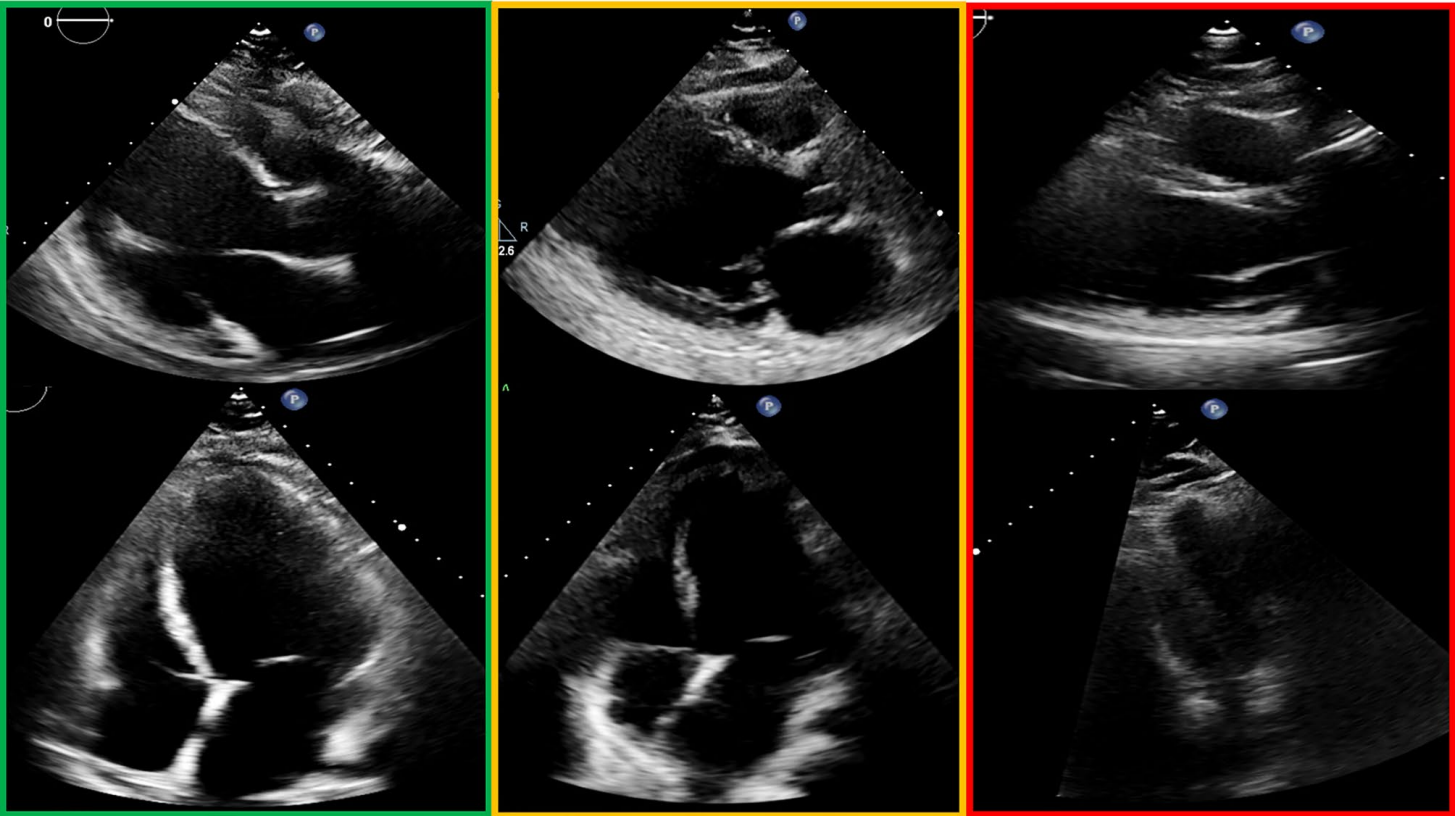
Representative transthoracic images at various stages of Duchenne muscular dystrophy disease progression. Top panel - Parasternal long axis (PLAx); Lower panel –Apical four-chamber (A4Ch); Left panel (green): 17-year-old patient at time of transition from paediatric care. Not yet requiring non-invasive ventilation; Middle panel (orange): 19-year-old patient with previous corrective spinal surgery for scoliosis. Not yet requiring non-invasive ventilation; Right panel (red): 36-year-old patient with prior scoliosis surgery. Established on non-invasive ventilation. The images demonstrate the progressive deterioration of image quality in the setting of disease progression and the establishment of non-invasive ventilation

Preserving left ventricular systolic function for as long as possible in DMD remains a key determinant of longer term survival [18]. Serial cardiac imaging is therefore essential, not only to monitor the trajectory of left ventricular systolic decline, but also to guide timely optimisation of HF therapy. However, DMD patients are subjected to the inherent limitations of TTE, including variable acoustic windows and reduced reproducibility as patients age and lose mobility (see Fig. 1). Additionally, TTE cannot provide tissue characterisation, such as on the presence of myocardial oedema or focal or more diffuse fibrosis. It is now established that the presence and extent of late gadolinium enhancement on cMRI, for example, herald progression to left ventricular dysfunction in the early stage of cardiac involvement and correlate more closely with risk of ventricular arrhythmias and the onset of overt cardiac failure at more advanced stages of cardiomyopathy than ejection fraction [19, 20]. Furthermore, the finding of changes in myocardial tissue components allows the detection of heart involvement in DMD at an even earlier stage than by TTE, and the range of measures it provides retains reproducibility and sensitivity to change as cardiomyopathy progresses [21, 22]. Imaging evidence of cardiac involvement allows an opportunity to escalate medical therapy, including the addition of mineralocorticoid receptor antagonists (MRAs), sodium glucose co-transporter II (SGLT2) inhibitors, or substitution of an ACE-I by Sacubitril/Valsartan when appropriate. Furthermore, knowing that a patient will develop LVSD at some point, patients and family members/carers are understandably keen to know how the patient’s heart is ‘doing’ serially as that information relates to their prognosis. Providing this information also reinforces the need for patients to take HF medications consistently.

## Unanswered questions and the need for further studies

The assumption inherent in much of the literature on the trajectory of decline in LV function in DMD is that it proceeds at a consistent predictable annual rate [23–25]. While this is probably the case for some patients, in others the deterioration occurs in acute, unpredictable, and sometimes even symptomatic episodes, triggered by random external influences [26–28]. It remains to be determined whether DMD has quite different cardiac phenotypes, explained perhaps by genetic or epigenetic patient differences, or whether the rate of decline is typically uniform but can be aggravated by factors such as viral illness, circulating promoters of inflammatory or adrenergic stress. If it were established that there are different cardiac phenotypes in DMD, it would be a significant confounder in studies aimed at establishing the true effectiveness of any medical intervention, for example. The uncertainty has implications for cardiac imaging schedules in patients with DMD also. For example, repeating assessments at fixed intervals would be sufficient to detect gradual changes in a timely manner, whereas abrupt changes would require imaging to be repeated in the context of the deterioration so that therapies could be intensified to aid re-stabilisation and limit the extent of permanent damage.

The natural history of cardiac function in DMD remains to be fully understood. To assess the true effectiveness of medical therapy, these cardiac “phenotypes” require better understanding, along with an understanding of the optimal point in time to commence these therapies. The concept of stepwise rather than smooth deteriorations in left ventricular function also challenges the validity and appropriateness of the current age-based recommendation to simply deploy cardioprotective medications prophylactically *‘no later than10-years of age’* [9]. That also requires further study. Cardiac imaging will undoubtedly hold the key to answering these key questions.

## Review of echocardiography left ventricular assessment methods

The BSE and other societies have published and regularly updated guidance on how TTE should be performed and which measurements should be routinely reported [29–31]. To understand how these apply to DMD, a comprehensive literature review of left ventricular systolic assessment in DMD was conducted. The search strategy, methodology, and narrative summary of the included studies are provided in the supplementary datafile with an overview of the findings shown below. However, this does not constitute a graded systematic review, hence recommendations that follow are based primarily on consensus expert opinion. This distinction is important because the heterogeneity of the available echocardiographic literature, variable quality of typically retrospective studies and a small number with methodology relevant to the current practice of echocardiography mean that the level of evidence available on which to base a systematic review is low. Indeed, well-designed long-term prospective natural history studies are rare in this patient cohort.

Twenty studies [22, 32–50] were identified, which included 853 participants (age 0–26.5 years), of whom 532 had genetically confirmed DMD. The most frequently assessed parameters were two/three dimensional (2D/3D) left ventricular ejection fraction (LVEF), 2D global longitudinal strain (GLS), and the myocardial performance index. Good intra and interobserver reproducibility was reported for LVEF and GLS. Across studies, there was a consistent lack of robust paediatric reference ranges and validated cutoff values for many proposed early markers of LVSD. The evidence base was heterogeneous, and several measurements could not be reliably obtained in all patients as the acoustic windows deteriorated with disease progression. Among all parameters, GLS and regional wall motion assessment showed the most consistent early abnormalities. GLS is a more sensitive indicator of early LVSD and is typically reduced at a time when LVEF and left ventricular fractional shortening (LVFS) are still within normal ranges. Several studies have shown an accerelated decline in LVSD in boys with DMD which begins with measures diverging from healthy peers from around seven years of age. These findings suggest that GLS can detect subclinical myocardial involvement earlier than global systolic indices; however, the absence of universally accepted paediatric reference values and validation of what constitutes a clinically significant difference limits its use as an absolute measure on which to make decisions about therapy. However, comparing the same result serially in the same patient over time allows detection of change. Other parameters such as mitral annular plane systolic excursion, myocardial performance index, tissue Doppler Imaging velocities, and dyssynchrony indices were variably abnormal but are constrained by methodological limitations, inconsistent reproducibility, and insufficient normative data.

In summary, no single TTE parameter beyond LVEF or LVFS currently has validated sensitivity, specificity, or reference standards sufficient to independently mandate treatment initiation. Nevertheless, because global left ventricular systolic indices are late markers of DCM in patients with DMD, early abnormalities in GLS or regional wall motion changes should prompt careful clinical review and consideration of initiating of HF therapies in line with contemporary guidelines. Normal reference ranges for diastolic function indices, tissue-Doppler Imaging velocities, mitral annular plane systolic excursion, and global longitudinal strain have not yet been established for children. For this reason, each patient’s baseline measurements should serve as the anchor for interpreting serial changes. This limitation underscores the value of a multimodality surveillance strategy, incorporating periodic cMRI alongside TTE, to enhance sensitivity in the detection of early myocardial involvement, further deterioration, and response to therapy.

## Practical issues to be consider

**Fig. 2:**
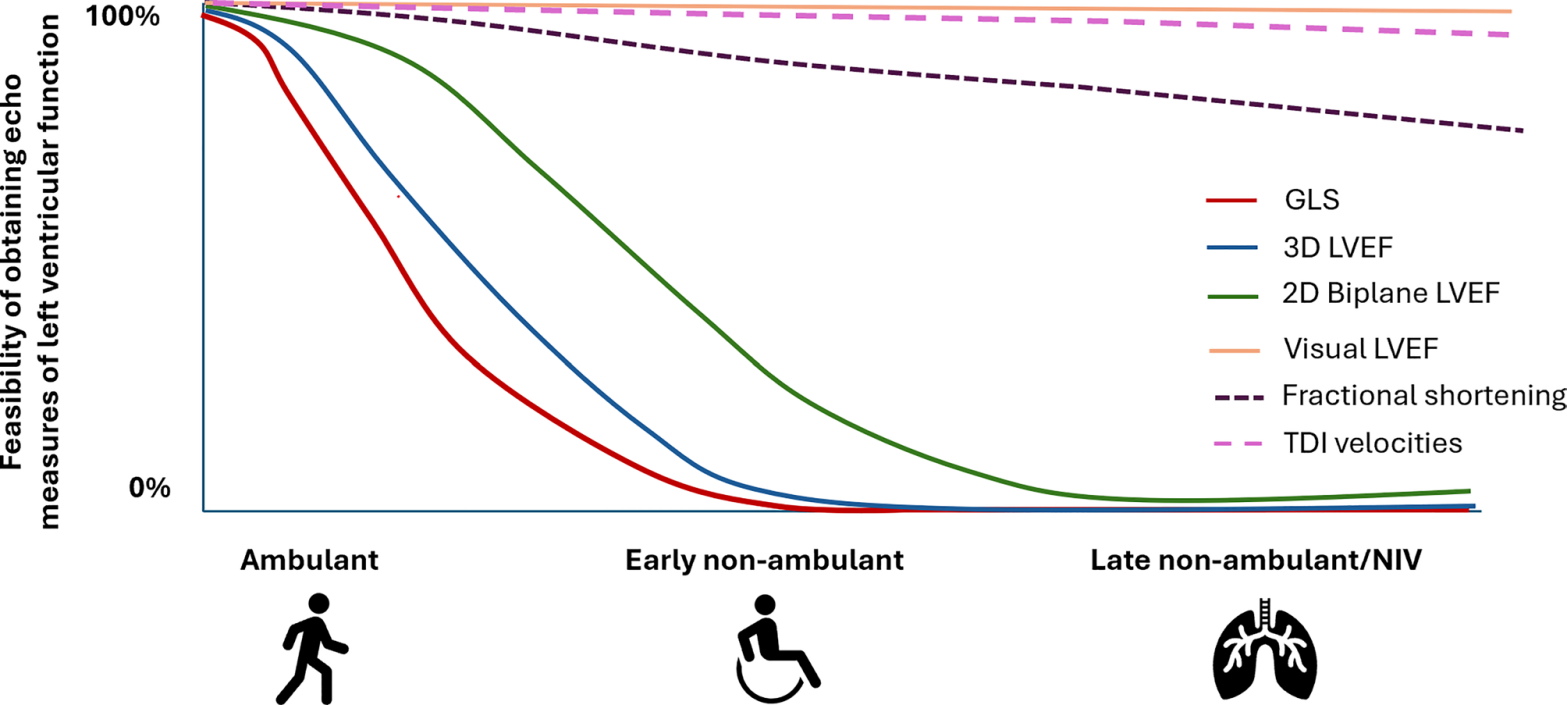
Feasibility of obtaining echocardiography measurements of left ventricular systolic function throughout the phases of Duchenne muscular dystrophy. LVEF: left ventricular ejection fraction, TDI: tissue Doppler imaging

Strengths and limitations of the various TTE parameters in DMD patients are summarised in Table 1 and Fig. 2. Obtaining a comprehensive set of TTE parameters poses challenges in patients with DMD, and the nature of the difficulties changes with the age and severity of the condition over time. Several factors need to be considered in the provision of TTE services for these patients, including the physical environment in which the scanning is performed and the possible need for a hoist or other equipment (e.g. if wheelchair reliant patients are to be moved to a couch to improve acoustic windows). A longer timeslot than usual may need to be scheduled, and best results will normally require echocardiographers with experience in scanning patients with muscular dystrophy. Considerations about performing a TTE and what TTE measurements are possible varies in patients with DMD and broadly relate to three stages of the condition (1): initial diagnosis and ambulatory (2), early non-ambulatory, and (3) late non-ambulatory stages. Feasibility of performing TTE measurements specific to LVSD are shown in Fig. 2 and are based on clinical experience and expert consensus opinion.

**Table 1:** Transthoracic echocardiography assessment parameters

Measurement	Explanatory note		Advantages	Disadvantages
2D and 3D Left ventricular ejection fraction (LVEF) N.B. BSE minimum dataset recommendation	Volume of blood ejected from the LV during systole, expressed as a percentage in comparison to LV diastolic blood volume. Taken from tracing the LV endocardial border in the A4C and A2C views at end-diastole and end-systole. Normal range (2D LVEF): >55%. An LVEF within the range of 50–55% should be considered abnormal in DMD patients	.	2D LVEF is well validated, has value in predicting poor outcomes. 3D LVEF is highly feasible and reproducible [12].	2D LVEF is reliant on adequate 2D images. Affected by preload and afterload. Inter-observer variability. 3D LVEF is reliant on adequate 2D images. Requires additional resources (3D transducer), knowledge and training. No current standardised normal reference ranges for 3D LVEF [12].
Tissue Doppler Indices (TDI) *N.B. BSE minimum dataset recommendation*	Velocity of myocardial movement throughout the cardiac cycle. Taken from the A4C. Normal mean mitral annular S velocity varies with age: 20–40 years ≥ 6.4 cm/s.		Easily obtainable and highly reproducible. Independent of 2D image quality.	Assumes function in regions assessed is reflective of entire ventricle and doesn’t account for RWMAs [12].
Global longitudinal strain (GLS) *N.B. BSE minimum dataset recommendation*	Myocardial deformation analysis of sub-endocardial longitudinal oriented LV fibres. Taken from A4C, A2C and A3C views. Normal range: >−16%.		Most reliable and clinically relevant for sub-clinical reductions in LV systolic function. Less angle dependent and allows tracking throughout the cardiac cycle.	Accuracy depends on 2D image quality. Requires additional knowledge and training. Influenced by pre and after load. Normal ranges can vary with age, gender and race [51]. If more than two segments in any one view are not adequately tracked, GLS becomes unreliable [52].
Fractional shortening (FS)	Percentage change in left ventricular dimensions between systole and diastole. Taken from 2D assessment of the basal LV regions in the PLAX view. Calculation: LVEDD - LVESD/LVEDD) x 100. Normal range: 25–45%.		Easily obtainable. Strongly correlated with left ventricular ejection fraction.	May overestimate or underestimate LV systolic function in the setting of RWMAs. Is affected by preload and afterload. Can be inaccurate if ventricular geometry is abnormal.
Mitral annular plane systolic excursion (MAPSE)	Vertical displacement or longitudinal motion of the mitral valve annulus during systole. Taken from M-mode assessment of the mitral valve annulus using the A4C view. Normal range > 13 mm.		Independent of 2D image quality. Easily obtainable and a reliable measurement that correlates well with LVEF [53].	Unable to detect RWMAs. Is angle dependent. Can be inaccurate in the setting of mitral valve disease, mitral valve annular calcification or mitral valve intervention [54].
Myocardial performance index (MPI)	Numeric value derived from the sum of left ventricular isovolumetric contraction and isovolumetric relaxation divided by total ejection time. Taken from pulsed wave Doppler or tissue Doppler indices of the mitral value annulus in the A4C view. MPI: isovolumetric contraction time (A) + isovolumetric relaxation time (B)/ejection time (B). Normal range: 0.39 ± 0.05.		Not reliant on high quality 2D images. Can be assessed by PW Doppler and TDI. Is independent of heart rate, preload and afterload. Is a reproducible and reliable measurement [55].	No defined normal “cut-off” for clinically meaningful LV abnormalities in DMD patients [56].

A4C: Apical four chamber, A2C: Apical two chamber, A3C: Apical three chamber, DMD: Duchenne muscular dystrophy, LV: Left ventricle, LVEDD: Left ventricular end diastolic dimension, LVEF: Left ventricle ejection fraction, LVESD: Left ventricular end systolic dimension, MPI: myocardial performance index, PLAX: Parasternal long axis view, PW: Pulse wave, RV: Right ventricular, RWMA: regional wall motion abnormalities, TDI: Tissue Doppler Index

### Initial diagnosis/ambulatory phase

In young children at initial diagnosis, patients are ambulant and can typically be scanned in the usual optimised positions on a TTE couch. Key challenges in scanning very young children apply, including a lack of co-operation and anxiety, often requiring distraction techniques and parental involvement. Additional factors may also impact the completeness of the examination, including the presence of severe autism or attention deficit hyperactivity disorder, both of which can be part of the DMD phenotype [57].

TTE image quality in these patients is adequate, and on-axis images can be obtained, allowing a full examination and comprehensive reporting.

### Early non-ambulatory phase

When a DMD patient becomes non-ambulant and wheelchair-reliant, the TTE is usually performed in the wheelchair. Most TTE scanning rooms will not allow for the use of a hoist to allow the child or young adult to be transferred safely from the wheelchair to TTE couch. At this stage of DMD, adequate images are generally still obtainable in the parasternal long-axis which are still usually on-axis, while apical views may not be obtainable or are often off-axis and foreshortened, due to the presence of chest wall deformities and/or scoliosis. By this stage of disease progression, the ability to perform GLS, 2D or 3D-derived LVEF is significantly compromised and presents a barrier to monitoring natural history, disease progression, and the effectiveness of any interventions.

### Late non-ambulatory phase

In patients at the more advanced stages of DMD, obtaining any measurements accurately by TTE may not be possible. In addition to constraints encountered in the early non-ambulant group, most patients will be using non-invasive ventilation, further impacting image quality and patient positioning. Although rarely used in practice, left ventricular opacification studies using contrast agents may enhance imaging to improve detection of left ventricular chamber borders and allow more accurate LVEF calculations. By this stage, the lack of image quality means that interpretation and measurements are usually qualitative with an associated increased inter-operator variability when compared to studies undertaken earlier in the disease.

## Summary of echocardiography recommendations

**Fig. 3:**
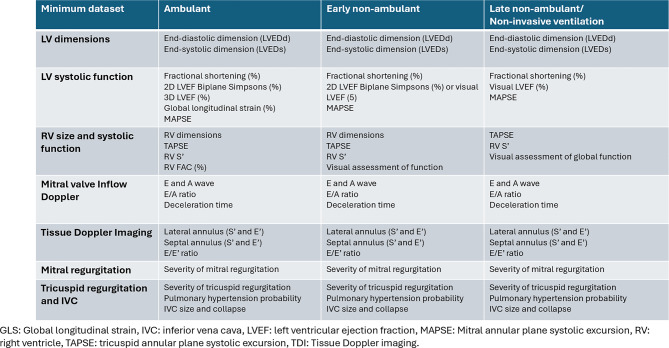
Minimum echocardiography dataset for ambulant, early non-ambulant and late non-ambulant/non-invasive ventilation stages

The following recommendations are based on an extensive review of the available literature and on the consensus opinion of clinicians and echocardiographers with expertise in TTE imaging and managing patients with DMD (see Fig. 3):

### Recommendation 1 – Baseline information required prior to undertaking the echocardiogram

Where the examination is not the patient’s first TTE a comprehensive TTE report should be available from the previous study, and particularly if the study has been performed in another centre. Previous images should be available to allow direct visual comparisons of the image quality and findings. This is especially important at the time of transition from paediatric to adult services, as this is often when changes in left ventricular function occur, mandating changes in cardiac management.

### Recommendation 2 – Initial diagnosis/ambulant phase

In the ambulant, paediatric population a comprehensive range of TTE measurements should be obtained and reported to provide the baseline against which future changes will be assessed (see Fig. 3). This should adhere as closely as possible to the BSE minimum dataset recommendations [11].

### Recommendation 3 – Non-ambulatory phase

When DMD patients become non-ambulant and wheelchair reliant, it is usual for the TTE to be completed with the patient sitting in their wheelchair. LVFS should be obtained and may be sensitive in identifying left ventricular dysfunction in the basal inferolateral segments of the left ventricle. This is the region typically first affected by this form of cardiomyopathy. Left ventricular systolic function should be assessed using quantitative methodologies in the first instance and should ideally include both 2D and 3D LVEF. As imaging quality declines, this hierarchy of left ventricular systolic function measurements may not be feasible or reliable. Under those circumstances a visual LVEF should be provided, although it should be made clear on the TTE report that the LVEF value is qualitative rather than quantitative. A comment should be made on regional wall motion abnormalities and lateral and septal Tissue Doppler Imaging systolic velocities whenever possible (see Fig. 3).

### Recommendation 4 – Contrast-enhancing agents

In the setting of having only limited/non-diagnostic TTE images, use of left ventricular opacification contrast-enhancing agents should be considered to improve endocardial definition and facilitate measurement of global left ventricular function. However, this should be undertaken on an individualised basis, as some left ventricular opacification contrast agents are not licenced for use in the patients under 18 years old.

### Recommendation 5 – Alternative imaging modalities

It should be stated clearly on the report when echocardiographers are no longer able to provide accurate measures of global left ventricular systolic function. The TTE report should recommend using another imaging modality (i.e. cMRI, cardiac computed tomography or radionuclide ventriculography).

## Conclusion

In patients with DMD, TTE provides detailed information about the extent of cardiac involvement and facilitates monitoring of the progression of the condition over time through serial assessments and a range of TTE measurements. This information helps clinicians to determine when to introduce conventional HF medications and when to increase the potency of the medical regime. Each individual TTE assessment should ideally comply with the recommended BSE minimum dataset. To enable valid comparisons of cardiac status over time, the same key measures should be obtained at each assessment. Echocardiographers need to recognise the extent to which image quality deteriorates over time in patients with DMD, particularly after patients lose ambulation, become wheelchair reliant, and cannot be positioned optimally.

## Electronic supplementary material

Below is the link to the electronic supplementary material.

Supplementary material 1

## Data Availability

The data that support the findings of this manuscript are available from the corresponding author upon reasonable request.

## Abbreviations

ACE-I: Angiotensin-converting enzyme inhibitor
BSE: British Society of Echocardiography
cMRI: Cardiac magnetic resonance imaging
DCM: Dilated cardiomyopathy
DMD: Duchenne muscular dystrophy
GLS: Global longitudinal strain
HF: Heart failure
LVEF: Left ventricular ejection fraction
LVFS: Fractional shortening
LVSD: Left ventricular systolic dysfunction
MRA: Mineralocorticoid receptor antagonists
SGLT2: Sodium glucose co-transporter II
UK: United Kingdom
2D: Two dimensional
3D: Three dimensional
