# Supplementary material for: Echocardiography in duchenne muscular dystrophy: a call for consistency and standardisation of reporting
Source: Echo Res Pract. 2026 Aug 3;13:28. doi: 10.1186/s44156-026-00130-6 (PMC13430808; doi:10.1186/s44156-026-00130-6)
Supplement: Supplementary file 1 — Supplementary material 1 [file 44156_2026_130_MOESM1_ESM.docx]

**Supplementary Data File**

**Literature review details**

A comprehensive literature review on the assessment of left ventricular (LV) systolic function in patients with DMD was conducted in December 2025. The process of study identification is summarised in Figure 1. Searches were performed in EMBASE, Medline, and PubMed for all study types published between January 2000 and December 2025. This timeframe was selected to capture evidence most relevant to modern TTE technology and to align with the evolution of contemporary TTE guidelines, ensuring that the findings reflect current clinical practice.

Electronic searches were complemented by manually searching all reference lists of identified studies and relevant reviews for additional studies. MeSH-related keywords such as “Duchenne muscular dystrophy” AND “echocardiography” OR “echocardiogra*” AND “left ventricle” OR “left ventric*”, as well as their common synonyms were included. Restrictions involved non-English language, animal studies, conference abstract, case reports / series, letters, editorials and reviews. Data extraction was undertaken by SB and LW using a standardised data extraction form. The following information was extracted from eligible studies: First author, year of publication, setting, study design, inclusion and exclusion criteria (where relevant), participant numbers, participant age range, echocardiography parameters assessed, reproducibility, sensitivity, ease of obtaining echocardiography measurements (see supplementary data file tables one and two). The following is a narrative summary of evidence provided by the literature review.

**Figure 1.** PRISMA flow diagram for study identification.

**Identification of studies via databases and registers**

Studies removed *before screening*:

Duplicate records removed (n = 350)

Records removed for other reasons: (n = 0)

Studies identified from*:

OVID (EMBASE and MEDLINE) (n = 778)

PUBMED (n = 578)

**Identification**

Studies excluded**

(n = 932) due to:

1. Non echocardiographic studies
2. Non DMD patients
3. No assessment of left ventricular systolic function

Studies screened

(n = 1006)

Studies sought for retrieval

(n = 70)

Studies not retrieved

(n = 0)

**Screening**

Studies excluded:

Non DMD patients (n = 6)

Non left ventricular systolic function assessment (n = 28)

Article with no original data (n= 5)

Outdated echocardiography techniques (n = 3)

Review articles (n = 5)

No access to articles and insufficient data in abstract (n = 3)

Studies assessed for eligibility

(n = 75)

Studies included in review

(n = 20)

**Included**

A total of 20 studies^22,27–45^ were identified. These included 853 patients, of which 532 had a genetically confirmed diagnosis of DMD. The age of included patients ranges from 0 - 26.5 years. In the seven studies that reported gender, 100% were males. There were 12 prospective studies^27,30–33,36,39–41,43,45^ and 6 retrospective studies.^22,29,35,37,42,44^ In the remaining 2 studies,^34,38^ the study design was not clear. In the studies that included a matched healthy control group, ^22,27,29,31–35,37–39,41,42,45^ patients were typically matched by age.

Multiple TTE parameters were assessed, included LVFS, two dimensional (2D) and three dimensional (3D) LVEF, 2D and 3D global longitudinal strain (GLS), LV dyssynchrony , mitral annular planar systolic excursion (MAPSE), myocardial performance index (MPI), regional wall motion index (RWMi), tissue Doppler imaging (TDI), E/A ratio, E wave deceleration time and LV dimensions and LV volumes. The most common TTE parameters employed were 2D/3D LVEF, 2D GLS, and MPI. Where assessed, 2D LVEF and GLS were assessed in “all patients”, ^22,27,32,34^ or “nearly all patients”.^41^ Good intra-observer and interobserver variability were also reported for 2D and/or 3D LVEF, ^27,30,39–41^ 2D and/or 3D GLS. ^30,35,37,39,41,45^

Two studies only included young male patients (mean age 9.1±2.8 years^39^ and 8.8±1.9 years) and the reported LVEF of DMD patients was comparable to that of healthy, age-matched controls. However, a significant decrease in GLS was observed prior to evidence of LV-remodelling or reductions in global measures such as LVEF or LVFS .^27^ An inverse relationship between age and GLS was noted in patients with DMD, ^27,31,39,45^ and the difference in GLS between DMD patients and healthy controls was most pronounced from 8 years old.^45^ LVEF was found to decrease to below 55% in DMD patients after 16 years old.^31^ When LVEF was within normal limits, GLS was noted to be a useful TTE parameter in detecting early LVSD ^30,31,33,34,37,41,45^ particularly as it was found to be significantly lower in DMD patients in comparison to healthy controls.^27,35,39,42,45^ A decrease in LVFS was found to occur prior to a decline in LVEF^37^ and was associated with the onset of DCM. ^22^ However LVFS was found to remain within normal limits despite impaired LV infero-lateral radial strain,^33^ was not associated with lateral MAPSE measurements^44^ and misclassified approximately 20% of patients with either normal or abnormal function.^40^ When compared to healthy controls, DMD patients presented with greater levels of LV dyssynchrony,^32^ lower TDI velocities,^38^ abnormal E/A ratios and lower E’ velocities.^42^ Baseline abnormalities in diastolic parameters in DMD patients were also associated with the development of DCM.

E/A ratios and lower E’ velocities.^42^ Baseline abnormalities in diastolic parameters in DMD patients were also associated with the development of DCM.

**TABLE ONE:** Data extraction - Study design, participant characteristics and study inclusion criteria.

| **Study ID** | **Study design; Country; Year** | **Total population** | **Age (years)** | **% Male** | **Participant inclusion criteria** |
| --- | --- | --- | --- | --- | --- |
| Amedro 2019 | Prospective; case control study; multicentre: France and Belgium; 2015-2016. | 108 (36 with DMD) | 0-17 | 100 | Patients with confirmed DMD and age matched healthy controls |
| Cho 2018 | Retrospective; case control study; single centre; Republic of Korea | 39 (13 with DMD) | 6-12 | Not stated | Patients with confirmed DMD and age matched healthy controls |
| Cirino 2018 | Prospective; cohort study; single centre; Brazil; 2011-2016 | 14 | 2-19 | 100 | Patients with confirmed DMD |
| Clavero-Adell 2024 | Prospective; case control study; single centre; Spain; 2020 | 38 (16 with DMD) | 4.4-26.5 | Not stated | Patients with confirmed DMD and age matched healthy controls |
| Lanot 2022 | Prospective case control study; multicentre; France and Belgium; not clear | 75 (25 with DMD) | 0-18 | Not stated | Patients with confirmed DMD and age matched healthy controls |
| Markham 2006 | Retrospective; case control study; single centre; US; 2001-2003 | 26 | 5-14 | 100 | Patients with confirmed DMD aged under 15 years with no signs or symptoms of heart failure and normal heart structure and function in normal sinus rhythm |
| Miyazaki 2008 | Prospective; case control study; single centre; Japan; 2007-2008 | 24 (12 with DMD) | 22.8±4.0 | 100 | Patients with confirmed DMD and age matched healthy controls |
| Ogato 2007 | Not clear; case control study; single centre; Japan | 23 (13 with DMD) | 11-20 | Not stated | Patients with confirmed DMD and age matched healthy controls |
| Oreto 2020 | Retrospective; case control study; single centre; Italy, not clear | 56 (32 with DMD) | 6-9 | Not stated | Patients with confirmed DMD and age matched healthy controls |
| Prakash 2022 | Prospective cohort study; India; 2018-2020 | 38 | 7-15 | 100 | Ambulatory boys with confirmed DMD |
| Ryan 2013 | Retrospective; case control study; US; 2009-2010 | 63 | 5.6±0.2 | 100 | Patients with confirmed DMD and age matched healthy controls |
| Seth 2010 | Not clear; case control study; not clear; not clear | 45 (25 with DMD) | 7.2±1.3 | Not stated | Patients with confirmed DMD and age matched healthy controls |
| Shehta 2021 | Prospective; case control study; single centre; 2019-2020; Egypt | 56 (28 with DMD) | 9.14 ± 2.85 | Not stated | Patients with confirmed DMD and age matched healthy controls |
| Soslow 2016 | Prospective; cohort study; single centre; US; not clear | 28 | 14.7 | Not stated | Patients with confirmed DMD |
| Spurney 2015 | Prospective; case control study; multicentre; US; not clear | 48 | 8.7-17.9 | Not stated | Patients with confirmed DMD or Becher muscular dystrophy |
| Taqatqa 2016 | Retrospective; case control study; single centre; 2014-2015; US | 19 | 11±3.7 | 100 | Patients with confirmed DMD and age matched healthy controls |
| Tsuburaya 2014 | Prospective; cohort study; single centre; Japan; not clear | 7 | 14-40 | 100 | Patients with confirmed DMD |
| Webb 2020 | Retrospective; cohort study; single centre; US; 2013-2015 | 59 | 1-21 | Not stated | Patients with confirmed DMD |
| Yu 2019 | Prospective; case control study; single centre; China; not clear | 87 (56 with DMD) | 8.8±1.9 | Not stated | Patients with confirmed DMD and age matched healthy controls |

DMD: Duchenne muscular dystrophy,

**TABLE TWO:** Data extraction – Echocardiography parameters assessed, image quality, reproducibility, sensitivity, ease of obtaining echocardiography measurements and summary of findings.

| **Study ID** | **Echocardiography parameter** | **Patient mobility** | **Overall image quality** | **Reproducibility** | **Sensitivity** | **Ease of obtaining echocardiography parameter** | **Summary of findings** |
| --- | --- | --- | --- | --- | --- | --- | --- |
| Amedro 2019 | 2D LVEF, E/A ration, E wave deceleration time, E/e’, GLS | 17/36 patients were ambulant | Not stated | Intra-observer ICC: 0.77-0.78. Inter-observer ICC: 0.62-0.68 | Not stated | No missing data for GLS. | In children with DMD, GLS was significantly decreased before the onset of DMD-related cardiomyopathy. The inferolateral and anterolateral segments were the most impaired, especially in the basal area. Moreover, children with DMD presented a significant decrease in GLS with age. |
| Cho 2018 | 2D LVEF, GLS, E/A ratio, E wave deceleration time, MPI | Not stated | Not stated | All measurements except E wave deceleration time had good interobserver ICC: 0.85-0.97 | Not stated | Not stated | GLS lower, most marked in inferior inferolateral, E/E' and E' velocities lower |
| Clavero-Adell 2024 | 2D LVEF, GLS | 11/16 patients were ambulant | Not stated | Not stated | Not stated | Not stated | GLS abnormal from young age with linear decrease, LVEF starts to fall below 55% after 16 years of age |
| Cirino 2018 | 3D LVEF, GLS | 18/54 patients were ambulant | Poor - 3 patients excluded due to image quality | Good interobserver correlation (r2 .66, p=0.0081) | Not stated | Not stated | GLS was useful in the detection of early LVSD |
| Lanot 2022 | LV mechanical dyssynchrony - opposing wall delay | 11/25 patients were ambulant | 3/20 DMD patients excluded due to poor image quality | Interobserver ICC: 0.49-0.94, intraobserver ICC: 0.60-0.97 | Not stated | All parameters assessed in all patients | DMD patients show evidence of greater dyssynchrony |
| Markham 2006 | FS, E/A ratio and IVRT | Not stated | Good to excellent | Not stated | Not stated | All parameters assessed in all patients | Baseline FS was lower in patients who went on to develop DCM. Baseline abnormalities in diastolic parameters (E/A ratio, prolonged IVRT) were associated with the development of DCM |
| Miyazaki 2008 | Radial strain, FS | 0/12 patients were ambulant | Not stated | Radial strain inter and intra -observer variability: 5-6% | Not stated | Not stated | Radial strain is impaired in infero-lateral LV region despite FS being within normal limits |
| Ogata 2007 | Radial strain, 2D LVEF | Not stated | Not stated | Not stated | Not stated | All parameters assessed in all patients | Radial strain is impaired in infero-lateral LV region despite LVEF being within normal limits |
| Oreto 2020 | GLS, 2D LVEF | Not stated | 3/35 excluded due to poor images | GLS Intraobserver variability 6.8% | Not stated | Not stated | All standard measures lower in DMD. GLS lower in all age groups |
| Prakash 2022 | TDI, FS, 5/6 area-length LVEF | All patients were ambulant | Not stated | Not stated | Not stated | Not stated | No correlate between echo LVEF, FS or GLS and CMR derived LVEF |
| Ryan 2013 | 2D LVEF, total and segmental GLS | Not stated | 5% of patients excluded due to poor image quality | Total GLS intraobserver ICC 0.91, interobserver variability ICC: 0.88.  Segment GLS intraobserver variability ICC 0.53, interobserver variability ICC 0.88 | Not stated | Not stated | Total and segmental GLS affected before LVEF and FS become abnormal |
| Seth 2010 | TDI velocities at lateral mitral annulus | 15/25 patients were ambulant | Not stated | Not stated | Not stated | Not stated | TDI velocities lower in both ambulant and non-ambulant DMD compared with controls |
| Shehta 2021 | 2D LVEF, GLS | Not stated | Not stated | Intraobserver variability ICC: 0.93, interobserver variability ICC:0.89 | Not stated | Not stated | LVEF was lower in DMD versus controls but still within normal range. GLS significantly lower. Inverse correlation between age and GLS (p 0.027, r-0.4) |
| Soslow 2016 | FS at PSAX at papillary muscle level, LVEF (single-plan, A4C only and biplane, A4C + A2C), GCS | 4/28 patients were ambulant | 60-64% of studies rated as average or better  Image quality reduced with increasing age | Most reproducible measurements were FS, 5/6 area-length LVEF and 3D LVEF.  FS and A4C LVEF had low reproducibility. | Not stated | Not stated | FS and 5/6 area-length LVEF had best combination of reproducibility and correlation with CMR LVEF. FS and 5/6 length LVEF mis-classified approximately 20% as either normal or abnormal function.  37% of LV segments were not visualised to assess for LWMi |
| Spurney 2016 | 2D LVEF, FS, MPI, GLS (A3C) and CS (PSAX at mid papillary muscle level) | Not stated | Not stated | LVEF: 0.49(0.19-0.70)  FS: 0.63(0.42-0.77)  GLS: 0.90 (0.77-0.95) | Not stated | LVEF, FS and GLS was feasible in 47/48 patients. | GLS detected myocardial dysfunction in patients with normal LVEF. |
| Taqatqa 2016 | GLS | 6/19 patients were ambulant | Not stated | Not stated | Not stated | Not stated | Total and segmental GCS was lower in DMD patients compared to controls |
| Tsuburaya 2014 | 3D LVEF | 0/7 patients were ambulant | Not stated | Not stated | Not stated | Not stated | 3D LVEF correlated well with radionuclide angiocardiography |
| Web 2020 | MAPSE and FS | Not stated | Not stated | Good interobserver correlation (r2 .66, p=0.0081) | Not stated | MAPSE is feasible in comparison to fractional shortening | No association between lateral MAPSE and FS |
| Yu 2019 | 3D LVEF, 3D GLS | Not stated | 9.7% of patients excluded due to poor image quality | 3D GLS intraobserver variability ICC: 0.8 (95% CI: 0.628-0.942, p=<0.001), 3D GLS interobserver variability ICC: 0.815 (95% CI: 0.532-0.927, p=<0.001). | Not stated | Not stated | In children with DMD the LVEF was similar to controls, GLS was significantly lower, and the difference was more pronounced in group > 8 years of age |

A2C: Apical two chamber, A4C: Apical four chamber, CMR: Cardiac magnetic resonance, DCM: Dilated cardiomyopathy, FS: Fractional shortening, GLS: Global longitudinal strain, HF: Heart failure, LVDd: Left ventricular diastolic dimension, LVEF: Left ventricular ejection fraction, LVSD: Left ventricular systolic dysfunction, MAPSE: Mitral annular planar systolic excursion, MPI: Myocardial performance index, PSAX: Parasternal short axis, TDI: Tissue Doppler imaging.
